# The Effects of Feature Sizes in Selectively Laser Melted Ti-6Al-4V Parts on the Validity of Optimised Process Parameters

**DOI:** 10.3390/ma13010117

**Published:** 2019-12-26

**Authors:** Chinmay Phutela, Nesma T. Aboulkhair, Christopher J. Tuck, Ian Ashcroft

**Affiliations:** Centre for Additive Manufacturing (CfAM), University of Nottingham, Nottingham NG8 1BB, UK; chinmay.phutela@nottingham.ac.uk (C.P.); christopher.tuck@nottingham.ac.uk (C.J.T.); ian.ashcroft@nottingham.ac.uk (I.A.)

**Keywords:** additive manufacturing, titanium alloys, selective laser melting, microstructure, mechanical properties, tensile behaviour.

## Abstract

Ti-6Al-4V is a popular alloy due to its high strength-to-weight ratio and excellent corrosion resistance. Many applications of additively manufactured Ti-6Al-4V using selective laser melting (SLM) have reached technology readiness. However, issues linked with metallurgical differences in parts manufactured by conventional processes and SLM persist. Very few studies have focused on relating the process parameters to the macroscopic and microscopic properties of parts with different size features. Therefore, the aim of this study was to investigate the effect of the size of features on the density, hardness, microstructural evolution, and mechanical properties of Ti-6Al-4V parts fabricated using a fixed set of parameters. It was found that there is an acceptable range of sizes that can be produced using a fixed set of parameters. Beyond a specific window, the relative density decreased. Upon decreasing the size of a cuboid from (5 × 5 × 5 mm) to (1 × 1 × 5 mm), porosity increased from 0.3% to 4.8%. Within a suitable size range, the microstructure was not significantly affected by size; however, a major change was observed outside the acceptable size window. The size of features played a significant role in the variation of mechanical properties. Under tensile loading, decreasing the gauge size, the ultimate and yield strengths deteriorated. This investigation, therefore, presents an understanding of the correlation between the feature size and process parameters in terms of the microscopic and macroscopic properties of Ti-6Al-4V parts manufactured using SLM. This study also highlights the fact that any set of optimized process parameters will only be valid within a specific size window.

## 1. Introduction

The application of the Ti-6Al-4V alloy is known in diverse industries, such as aerospace, automobile, chemical, and medical ones [1,2,3]. This popularity is attributed to the low density-to-strength ratio, exceptional corrosion resistance, and biocompatibility of this ⍺ + β Titanium (Ti) alloy [4]. In Ti-6Al-4V, Ti is alloyed with 6 wt % aluminium (Al), which is an α-stabiliser, and 4 wt % vanadium (V), which is a β-stabiliser lowering the transus temperature [5]. Hence, Ti-6Al-4V exists as a mixture of α + β equilibrium microstructure at room temperature. Even though it is known for its high performance in various aspects, the industrial use of Ti-6Al-4V remains limited to specific applications due to its high cost. The major cost manifests from the processing steps involved in the manufacturing of Ti components, which require high energy inputs while creating a significant amount of waste [6,7,8]. Therefore, the need for alternative manufacturing methods is growing, and additive manufacturing (AM) is one of the top contenders.

AM plays an essential role in mitigating the aforementioned issue with conventional manufacturing of Ti-6Al-4V [9]. In any AM process, a computer-aided design (CAD) file is used as an input, and it is then primarily fabricated in a layered pattern [10]. Due to the layered approach, AM allows high design complexity freedom [11]. Apart from the well-established complexity advantage, AM permits minimal raw material wastage, except in the case of support structures [12]. This study focusses on the laser powder bed fusion (L-PBF) process, commonly known as selective laser melting (SLM) [13]. The operational concept of the technology can be found in [14]. The material flexibility and ability to produce components with high precision due to small beam diameter (typically 20–100 µm) lasers are some of the added advantages of SLM when compared to other metal-based AM technologies [15,16]. SLM has reached a point where nearly 100% (99.7% or more) dense parts can be fabricated [17]. SLM parts have shown evidence of possessing comparable or even often superior mechanical properties when compared with traditionally manufactured parts [18,19]. Complexity freedom of AM and the exceptional properties of Ti-6Al-4V opens the opportunity for numerous high-end applications [20,21]. For instance, the “buy to fly” ratio for conventionally produced large aerospace parts can be in range of 8–12:1 and sometimes as high as 30:1, whereas it is approximately 1.5:1 in the case of components manufactured by AM [22].

The increasing use of SLM, especially with Ti-6Al-4V, in high-value applications, necessitates the manufacturing process becoming highly consistent and reliable. However, defects, such as hydrogen porosity, lack-of-fusion pores, keyhole defects, cracks, and impurities, are common [23,24]. The significant influence of defects on the mechanical properties of Ti-6Al-4V parts is not unusual [25,26]. Hence, it is essential to analyse and suppress defect formation to ensure a reliable manufacturing system for large-scale industry acceptance. Gibson et al. [27] reported that melt pool size, layer thickness, scan speed, scan strategy, etc., are highly significant parameters to manufacturing high density components using SLM. Melt pool size and depth are majorly dependent on the energy absorbed by the powder bed when scanned by the laser [28]. Numerous studies have been performed to produce highly dense SLM parts by optimising the process parameters [29,30,31,32,33]. Gibson et al. [27] categorically listed the process parameters which affect the density, and hence the mechanical properties of SLM parts, including the laser power, scan speed, hatch spacing, layer thickness, and scan strategy. Process parameter optimisation for defect suppression and overall build quality in the case of Ti-6Al-4V SLM parts has also been extensively studied [34,35,36,37]. There are several studies also focusing on the microstructure and mechanical properties of SLM Ti-6Al-4V parts [38,39,40,41]. However, minimal studies have recognised the importance of the size of the features on the quality of these parts; i.e., how the size of a feature can influence the density, microstructure, and mechanical properties of a particular part due to variations in the thermal profile within these features during processing.

There have been a few studies that indicate how the overall microscopic and macroscopic quality of SLM parts can be size-dependent [42,43,44,45]. However, the current literature lacks a systematic study which reports on the influence of the size of a sample on the porosity and microstructure of as-built Ti-6Al-4V samples. Further, studies on the impacts of changes in size on the performances of stress-relieved Ti-6Al-4V specimens under tensile loading are scarce. Therefore, the current study aims at filling the research gap by attempting to explore the influence of the “size of features” on SLM parts’ densities, hardness values, microstructures, and mechanical properties.

## 2. Materials and Methods

Plasma atomized Ti-6Al-4V Grade 23 powder supplied by LPW Technologies (UK) was used in this study. The chemical composition of the powder is presented in Table 1. The powder size distribution was determined using a Mastersizer 3000 (Malvern, UK), which uses the laser diffraction method [46]. D10, D50, and D90 values for powder particles were 19.6, 31.5, and 49.1 µm, respectively. The powder particles had a spherical morphology with a few satellites attached to the particles.

All samples were manufactured with a Realizer SLM 50 system, equipped with a 100 W yttrium fibre laser, using a fixed set of process parameters. Samples were fabricated using 82.5 W laser power, 20 µs exposure time, 20 µm point distance, 90 µm hatch distance, and 40 µm layer thickness. These parameters were optimised for 5 mm cubes in prior research. A double scanning strategy, where each layer was scanned twice using alternate parallel scanning vectors, was employed. The process chamber was flushed with argon to attain an oxygen level below 0.5%.

**Figure 1:**
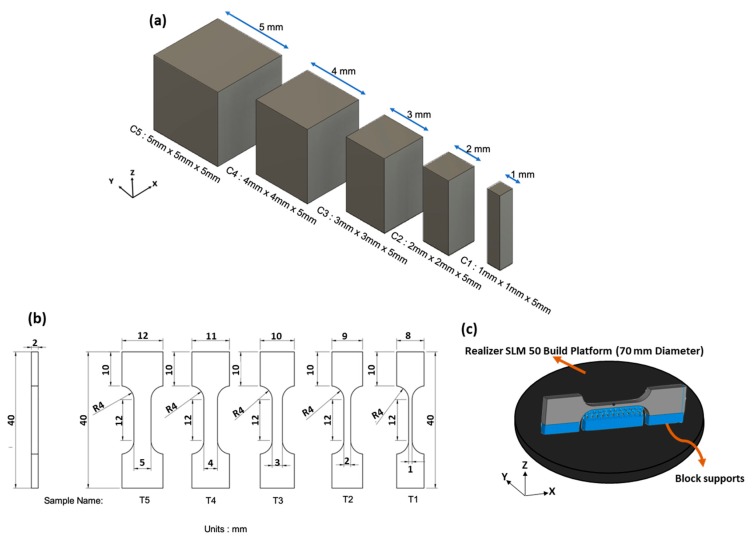
(**a**) C1–C5 samples with a fixed Z height of 5 mm and variation in length and width from 1 to 5 mm. Height was fixed as 5 mm to avoid poor handling of small samples. (**b**) CAD drawing of tensile samples (T1–T5) and their size parameters. (**c**) Representation of T1 sample oriented in Flat XZ direction.

Two sets of specimens were manufactured for the various tests conducted in this study (Figure 1a,b). Orthogonal planes of each sample category were defined as per ASTM F2921-11 [47]. Cuboidal samples C1–C5 were manufactured to analyse porosity and microstructure related changes. For each sample, three repetitions were fabricated in order to analyse one orthogonal plane per repetition for every size. This was done to attain statistical confidence in the data. All orthogonal planes (frontal YZ, lateral XZ, and horizontal XY) were analysed for porosity and microstructure using a Nikon Optiphot 100 (Nikon, Japan) optical microscope (OM) and image processing using Image J [48]. The polished samples were tested for micro-hardness using Buehler Wilson VH3100 vickers hardness tester (Buehler, Germany) with a 500 g load and 10 s dwell time.

Non-standard tensile bars (Figure 1b) were designed in order to fit in the Realizer system’s cylindrical build volume (70 mm diameter and 40 mm height). The tensile samples were built in flat XZ direction (Figure 1c). Depending on the gauge width variation from 1 to 5 mm, the name varied from T1 to T5, respectively. The naming convention defined in Figure 1 will be used hereafter. Block support structures were added to support overhang features in the tensile bars (Figure 1c). Three repetitions for each tensile bar were manufactured for statistical confidence.

All tensile specimens were stress-relieved at 730 °C for 2 h in argon environment. The samples were tested using an Instron 5969 universal testing machine (Instron, MA, USA). A black and white speckle pattern was applied to the samples to collect strain data using a video gauge. The crosshead speed was 2 mm/min, and tests were performed at room temperature. A Hitatchi TM3030 scanning electron microscope SEM (Hitatchi, Japan) was used for fractography analysis on the tensile specimens after the tests.

## 3. Results and Discussion

### 3.1. Influence of Size on Density, Microstructure, and Hardness of As-Built SLM Ti-6Al-4V Parts

**Figure 2:**
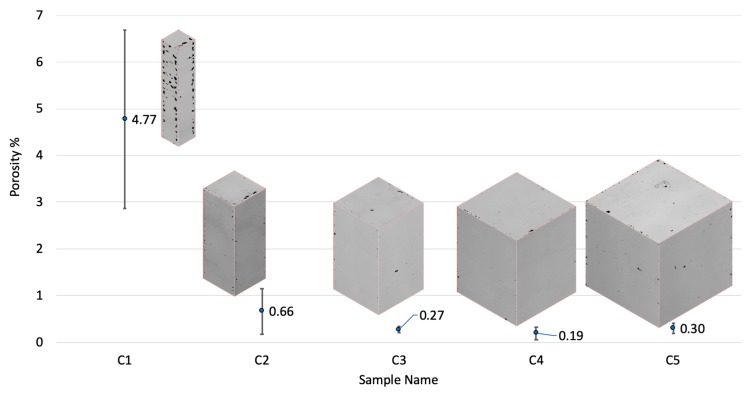
Variation of porosity percentage with respect to the size parameters of the samples. C1 had the highest porosity, and C4 had the lowest. Error bars in the plot represent the standard deviations of the values.

The 3D reconstructions of the cross-sectioned samples and their porosity percentages are presented in Figure 2. In the majority of the samples, defects preferentially aligned around the edges of the cross sections. This preferential alignment could be linked to the porosity emerging between the contour scan and core scan regions. This is in agreement with an earlier study by Yang et al. [49] for Al alloys.

In a study by Koutney et al. [42], Al alloy cubes with varying edge lengths were manufactured. The study reported that the porosity in the top plane of the cubes increased from 0.03% to 5.95% on changing the size from 5 to 13 mm, respectively. Analysing the size effect was, however, not the focus of that study. In another study by Dong et al. [43], an increase in porosity was reported with decreasing the gauge diameter of AlSi10Mg samples; porosity changed from 1.87% to 0.1% by increasing the gauge diameter from 1 to 5 mm, respectively. This agrees with our findings supporting the hypothesis that only a specific window of sizes can be manufactured with high density by using a fixed set of parameters. Once beyond this window, the process parameters will require readjustment. Based on the current results, we conclude that there was an increase in porosity with the decrease in sample size. To define an acceptable size window for a particular set of parameters, an extensive set of samples with size variation is required. It was noted that larger samples (C3, C4, and C5) had lesser variations in their porosities; however, smaller samples (C1 and C2) showed much higher density variations. Upon decreasing the sample size from C5 to C2, porosity increased by nearly 120%. The increasing porosity with size decrease could be attributed to the temperature distribution (thermal profile) intrinsic to the process. Sample C1 is the extreme case with porosity exceeding 4%, the defects in C1 being comprised of a mixture of keyhole and lack-of-fusion pores. However, lack-of-fusion defects dominated over the keyhole defects in majority of the cross-sections along all the orthogonal planes. Lack-of-fusion defects typically correspond to insufficient energy received by the metal powder, limiting the melting and fusion of layers and resulting in pores/voids with unsintered powder trapped inside the pores [23]. The high number of lack-of-fusion defects in C1 can be attributed to the fewer number of scan tracks as the size decreased, which might have led to insufficient energy inputs, resulting in poor melting and fusion of the powder.

**Figure 3:**
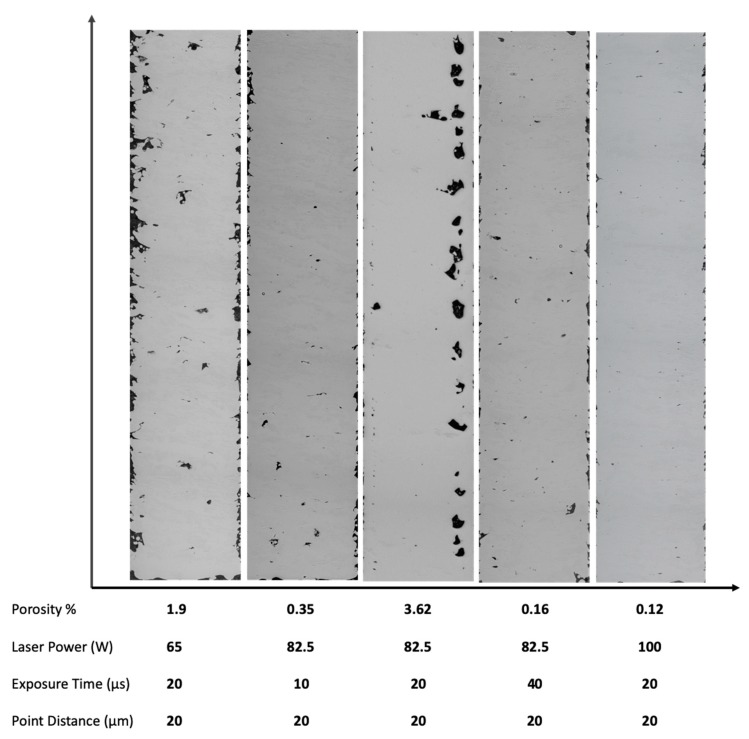
Changes in porosity percentages by varying laser current and exposure time process parameters. Original C1 samples had 3.62% porosity resulting from a 82.5 W laser and exposure time of 20 µs.

To confirm this hypothesis, we trialled four sets of process parameters (varying the laser power and exposure time) for C1 samples and analysed the density variation. As shown in Figure 3, upon increasing the laser power (i.e., increasing the energy density), porosity reduced significantly. Unexpectedly, upon reducing the laser power, internal porosity still reduced; however, the surface roughness increased significantly, leading to poor quality samples. It was also observed that on fixing the laser power and reducing the scan speed (still increasing the energy density using a different approach), porosity improvement was better than the case of increasing the scan speed. Conclusive proof requires further research on process parameter optimisation with small samples to deal with uncertainties. However, the current results still prove the insufficiency of the input laser energy in the original C1 samples. The study by Sun et al. [50] suggests that Ti-6Al-4V samples possess high density when manufactured using high laser power and low scanning speeds. This statement holds for C1 samples manufactured using a range of process parameters (Figure 3).

**Figure 4:**
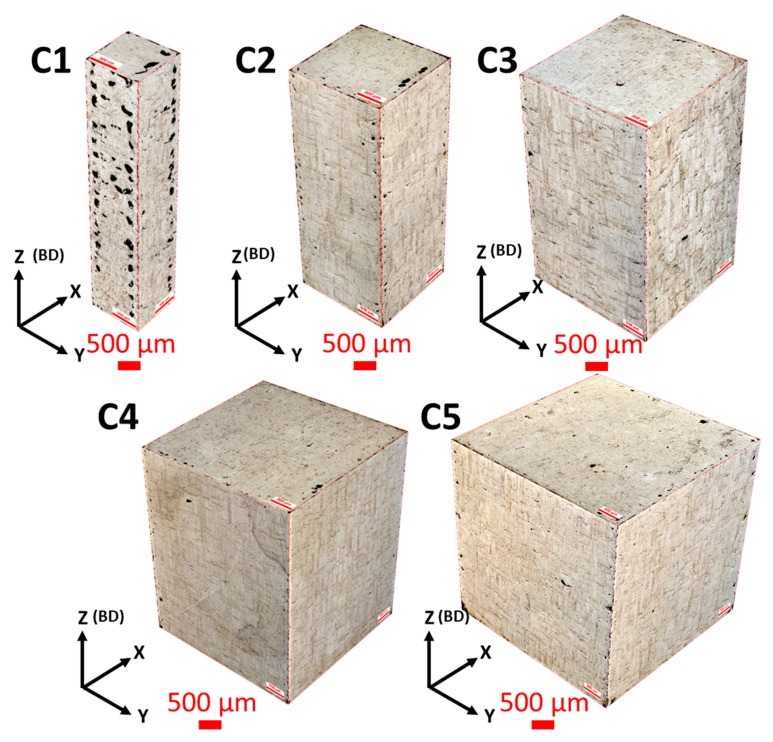
Optical micrographs representing the microstructures of samples C1–C5, showing the isometric reconstruction in the three orthogonal planes for each sample. BD: build direction.

**Figure 5:**
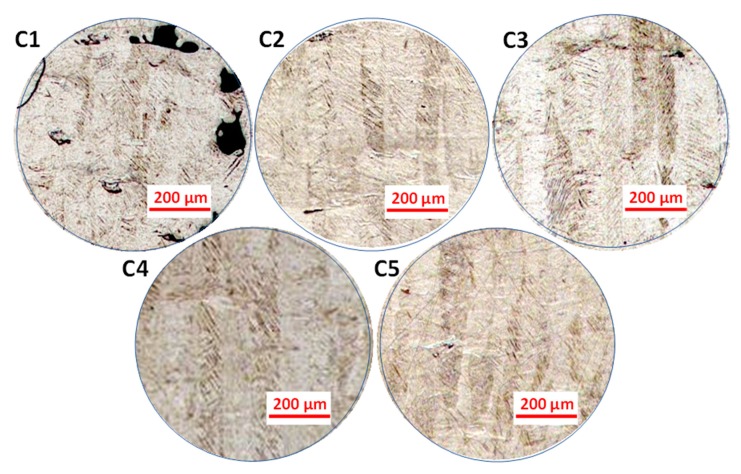
Optical micrographs representing the microstructures of the lateral XZ planes of C1–C5.

In terms of the metallurgical properties, studies show that the microstructure of SLM as-built Ti-6Al-4V comprises α’ martensitic phases. The α’ grains “grow within elongated prior β grains, which grow through the deposition of consecutive layers” [40,51]. In the samples analysed in this study, lateral and frontal planes comprised of fine α’ acicular grains with prior β grain boundaries are visible in the micrographs in Figure 4 and Figure 5. This is in agreement with the literature. β grains had a lenticular morphology, while α’ ones were organized within the prior β grain boundaries [52]. Top planes, on the other hand, revealed cross-sections of the β grains (Figure 4).

β columnar grains were present because solidification of Ti-6Al-4V in SLM takes place in the β phase field, and heat is mainly conducted away vertically [38]. The orientation of β grain boundaries has a high dependence on heat conduction direction when the laser is scanning a layer [40]. Therefore, with the double scanning strategy used in the current study, prior β grain boundary vectors were expected to be parallel to the build direction. This held for the samples in this study, with some misorientations observed in the smallest sample C1 (Figure 5). Thijs et al. [40] reported extreme dependence of the prior β grain boundary’s orientation on the scanning pattern, and hence, on the local heat transfer conditions in the powder bed; i.e., the thermal gradient. Therefore, the misorientations observed in C1 could be attributed to the limited local heat conduction in any particular direction resulting from insufficient energy input, as discussed in case of porosity variation due to change in size.

The β grain boundaries in samples C1–C5 spanned across tens of layers, with each layer being 40 µm in height. The β grain width was mostly consistent in all the samples, as ≈90 µm, which corresponded to the hatch distance used in the study. Table 2 lists the β grain size for each sample size. Therefore, the morphologies and sizes of the prior β grain boundaries in the as-built Ti-6Al-4V parts were not significantly affected within the acceptable size range, as also noted by Zhao et al. [45].

**Figure 6:**
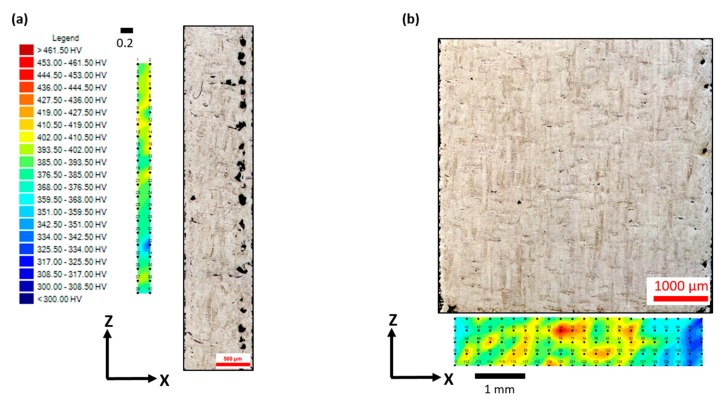
Micrograph representing the microstructure of sample C1 in (**a**) showing the prior β grains slightly misoriented alongside the corresponding micro-hardness profile. (**b**) The microstructure of sample C5 showing the prior β grains growing parallel to the build direction, along with the respective micro-hardness profile.

**Figure 7:**
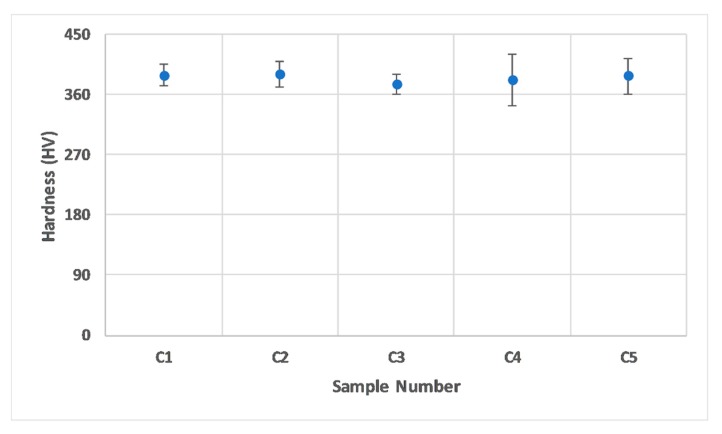
Average hardness values for samples C1–C5. Error bars in the plot represent the standard deviation values.

Figure 6 represents the micro-hardness profile map for samples C1 and C5. Figure 7, on the other hand, shows a plot for the overall hardness variation with respect to the sample size. There was no particular trend in hardness values with the change in size. Considering the error bars, hardness values for all the samples were comparable, and the two extreme samples (C5 and C1) had a rather close average hardness value (≈386 HV). This is comparable to the micro-hardness of Ti-6Al-4V reported in the literature, which typically varies between 340 and 500 HV [40]. Since the change in the sample size did not have a significant influence on the material’s microstructure, the hardness of the material was not affected. This is owing to the nature of hardness as a material property, which mainly corresponds to the microstructure and not necessarily to the defects to a certain extent. It is important to note that the pore fraction in a sample can influence the hardness of a material once it surpasses a critical limit [53,54], which is material dependent. For the case of Ti-6Al-4V within the range investigated in the current study, this was not substantial.

### 3.2. Influence of Size on Tensile Properties and the Fracture Mechanism of Stress Relieved Ti-6Al-4V

**Figure 8:**
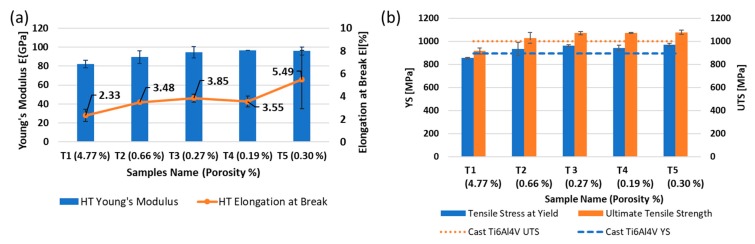
(**a**) Young’s modulus and elongation at break. (**b**) Tensile stress at yield and ultimate tensile strength of tensile samples. Dashed lines represent the properties for cast Ti-6Al-4V. The average porosity for each sample size is quoted between brackets next to the sample name on the x-axis.

The microstructure of as-built SLM Ti-6Al-4V might not be appropriate for the present applications of conventionally produced α + β alloy. This limitation stems from the as-built components showing poor ductility, even though the strength is usually noted to be higher than conventionally manufactured Ti-6Al-4V [55]. In order to maintain a balance between strength and ductility, various post-process treatments have been introduced in the past [39,56]. Figure 8 presents plots for the mechanical properties of tensile (T1–T5) samples.

By decreasing the gauge width of tensile samples from 5 to 1 mm, tensile stress at yield (YS), ultimate tensile strength (UTS), and Young’s modulus (E) experienced decreases of approximately 12%, 15%, and 14%, respectively. However, the elongation at break (El) was found to be more sensitive to change in size and decreased by ≈57.6%. The variation in the tensile properties can also be correlated to the amount of porosity in the samples by parallelism [57] and the gauge size [58]. Literature on the latter shows that the use of non-standard tensile test bars does not affect the strength significantly but can influence Young’s modulus and the elongation to break. However, the responses of these properties to variations in the gauge size are not consistent and can be material dependent, as demonstrated by these articles: [58,59]. The lowest values of porosity in samples T3–T5 granted them higher tensile properties. As the porosity content almost doubled in sample T2, the tensile properties of the material started deteriorating. An increase in porosity by almost 15 times in sample T1 resulted in the most significant deterioration in properties within the range investigated in this study. This suggests that the size of features had a significant effect on the mechanical properties of parts processed by SLM due to the correlation between the sizes of the features and the amount of porosity forming in the samples during processing using a fixed set of process parameters.

**Figure 9:**
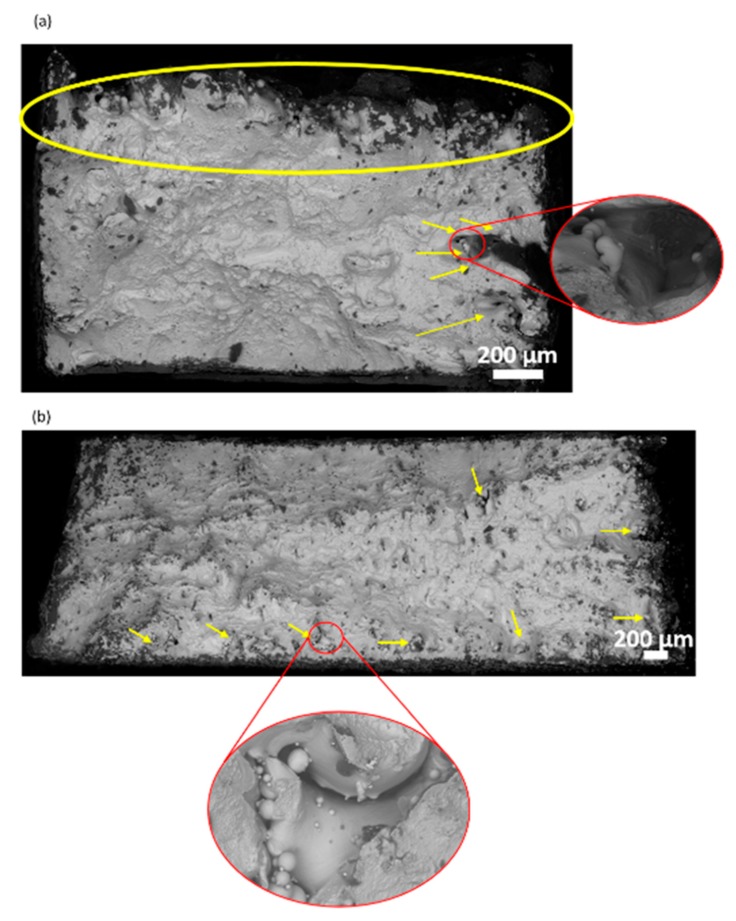
Fractography images of (**a**) T1 and (**b**) T5 tensile bars. Yellow regions mark the pores on the surface.

Figure 9 highlights terrace-like features in both T1 and T5 samples, also known as “layered fracture” [60]. The fracture surfaces had a relatively flat central portion and shear lips inclined at 45° to the loading direction. The images reveal that the most reasonable fracture mode for the Ti-6Al-4V SLM samples was inter-granular, which is in agreement with the discussion presented by Simonelli et al. [51]. Overall, fracture surfaces were composed of shallow dimples, cleavage facets, and open pores in both cases, suggesting a combination of brittle and ductile fracturing. The maximum porosity or the region with the most unfused powder particles was where the support structures were created during manufacturing. However, T1 possessed greater porosity than T5 when comparing their porosity regions per overall area of the sample’s surface (Figure 9).

Interestingly, in the results reported by Dong et al. [43] for AlSi10Mg samples, strength deterioration due to size variation showed a similar overall trend in the change of mechanical properties due to the change in features size to the case of Ti-6Al-4V samples used in the current study. This suggests that size of features had a significant effect on the mechanical properties of the parts processed by SLM, and that the magnitude of its influence is dependent on the set of optimised SLM parameters used. The current study shows a greater influence of change in size on mechanical properties and microstructure compared to the results reported by Zhao et al. [45] for SLM Ti-6Al-4V samples. Zhao et al. [45] manufactured samples in the form of cylinders which were machined down for testing. Therefore, the results might not wholly be comparable to the case of a sample solely manufactured additively without further machining. This is mainly because the thermal profile would be different in the case of printing small features directly (as has been shown in the current study) from printing large structures and then machining out a smaller feature. The former is more relevant to the AM community, since ultimately, SLM is a net shape manufacturing process. This is clearly supported by the porosity distribution presented in Figure 2, which shows that porosity in the smallest samples was mainly located near the edges of the samples. Had these samples been machined, the results may have been in agreement with what has already been published in the literature due to machining out the defected region. This research will also be more applicable to areas where using the design freedom in AM is of paramount importance, such as the work on fabricating latticed structures where part machining is difficult or impossible.

Considering the overall results, it is interesting to note that larger samples (T3–T5) displayed higher consistency in terms of E, UTS, and YS when compared to smaller samples (T1 and T2). A similar trend was also evident in porosity and microstructure variation in the as-built Ti-6Al-4V samples (C1–C5), as discussed earlier. Since hardness is fundamentally related to the yield strength of a material, the yield strengths of the samples with two extreme feature sizes were expected to be of similar scale in this study, when considering the hardness values of samples presented in Figure 7. Therefore, any variation in mechanical properties of the tensile bars with differences in gauge width can be considered dependent on the defect formation induced due to the change in size and not the material itself.

## 4. Conclusions

This study investigated the influence of feature size on the defect formation, microstructure homogeneity, and mechanical performance of Ti-6Al-4V parts produced by SLM. One of the motives behind this work was to assess the applicability of a fixed set of “optimised” process parameters to features of a wide range of sizes. Various parts were manufactured using a fixed set of parameters, originally optimized for 5 × 5 × 5 mm^3^ samples, as typically done in SLM, while varying the size between 1 and 5 mm. The results of this study confirmed the dependence of the quality of the samples on their size at a given set of process parameters. As the sample size decreased beyond a certain threshold, porosity increased significantly, resulting in poorer tensile properties (strength and ductility). The yield strength decreased by ~12%, ultimate tensile strength by ~15%, and elongation by ~56%, upon decreasing gauge width from 5 to 1 mm. The effects of the sample size on the microstructure and hardness were, however, negligible. The variation in porosity with sample size was attributed to the significant change in the thermal profile and gradients within the material during processing. Lack-of-fusion defects in the smaller samples was exacerbated by the low energy inputs, and increasing the energy density successfully reduced porosity in these samples. This shows that a tweak in the process parameters is necessary to cope with this phenomenon to ensure the fabrication of parts of reliable structural integrity for all feature sizes. Massive efforts in the research community are directed towards optimizing the process parameters for materials to be processed by SLM. However, without taking into account the influence of the feature size, as this study has shown, the applicability will be compromised, particularly when it comes to applications in the areas where topology optimized and latticed structures are of paramount importance, where standard optimized process parameters might not be reliable.

## Figures and Tables

**Table 1:** Chemical composition of Ti-6Al-4V Grade 23 powder provided by LPW.

Element	N	C	H	Fe	Al	V	Ti
Chemical Composition (wt %)	0.03	0.08	0.0125	0.25	5.5-6.5	3.5-4.5	Balance

**Table 2:** Prior β grain width in as-built Ti-6Al-4V samples (C2–C5).

Sample Name	Length	Width (µm)
C1	Order of millimeters	84.19 ± 1.76
C2	Order of millimeters	89.90 ± 8.04
C3	Order of millimeters	91.75 ± 6.18
C4	Order of millimeters	91.96 ± 9.42
C5	Order of millimeters	88.97 ± 6.19
